# Identification of nine microRNAs as potential biomarkers for lung adenocarcinoma

**DOI:** 10.1002/2211-5463.12572

**Published:** 2019-01-09

**Authors:** Zhi‐Peng Ren, Xiao‐Bin Hou, Xiao‐Dong Tian, Jun‐Tang Guo, Lian‐Bin Zhang, Zhi‐Qiang Xue, Jian‐Qing Deng, Shao‐Wei Zhang, Jun‐Yi Pan, Xiang‐Yang Chu

**Affiliations:** ^1^ Department of Thoracic Surgery Chinese PLA General Hospital Beijing China

**Keywords:** biomarker, DEmiRNA, LUAD, lung adenocarcinoma, microRNA, TCGA

## Abstract

Lung cancer is a leading global cause of cancer‐related death, and lung adenocarcinoma (LUAD) accounts for ~ 50% of lung cancer. Here, we screened for novel and specific biomarkers of LUAD by searching for differentially expressed mRNAs (DEmRNAs) and microRNAs (DEmiRNAs) in LUAD patient expression data within The Cancer Genome Atlas (TCGA). The identified optimal diagnostic miRNA biomarkers were used to establish classification models (including support vector machine, decision tree, and random forest) to distinguish between LUAD and adjacent tissues. We then predicted the targets of identified optimal diagnostic miRNA biomarkers, functionally annotated these target genes, and performed receiver operating characteristic curve analysis of the respective DEmiRNA biomarkers, their target DEmRNAs, and combinations of DEmiRNA biomarkers. We validated the expression of selected DEmiRNA biomarkers by quantitative real‐time PCR (qRT‐PCR). In all, we identified a total of 13 DEmiRNAs, 2301 DEmRNAs and 232 DEmiRNA–target DEmRNA pairs between LUAD and adjacent tissues and selected nine DEmiRNAs (*hsa‐mir‐486‐1*,* hsa‐mir‐486‐2*,* hsa‐mir‐153*,* hsa‐mir‐210*,* hsa‐mir‐9‐1*,* hsa‐mir‐9‐2*,* hsa‐mir‐9‐3*,* hsa‐mir‐577*, and *hsa‐mir‐4732*) as optimal LUAD‐specific biomarkers with great diagnostic value. The predicted targets of these nine DEmiRNAs were significantly enriched in transcriptional misregulation in cancer and central carbon metabolism. Our qRT‐PCR results were generally consistent with our integrated analysis. In summary, our study identified nine DEmiRNAs that may serve as potential diagnostic biomarkers of LUAD. Functional annotation of their target DEmRNAs may provide information on their roles in LUAD.

**Abbreviations**
AUC: area under the curve
DEmiRNA: differentially expressed microRNA
DEmRNA: differentially expressed mRNA
DOR: diagnostic odds ratio
FDR: false discovery rate
GO: gene ontology
KEGG: Kyoto Encyclopedia of Genes and Genomes
LUAD: lung adenocarcinoma
miRNA: microRNA
NSCLC: non‐small‐cell lung cancer
qRT‐PCR: quantitative real‐time PCR
ROC: receiver operating characteristic
SVM: support vector machine
TCGA: The Cancer Genome Atlas

Lung cancer is the most frequently diagnosed cancer and a leading cause of cancer‐related death worldwide 1. Approximately 4/5 diagnosed lung cancers are non‐small‐cell lung cancer (NSCLC), and lung adenocarcinoma (LUAD) is the most common histological subtype of NSCLC, accounting for ~ 50% of lung cancer 2, 3. Due to the lack of effective diagnostic tools, most LUAD patients are diagnosed at the later stages, and the 5‐year survival rate of LUAD has shown no significant improvement over the past few decades 4. Hence, it is essential to identify the novel, non‐invasive, and specific biomarkers for the diagnosis of LUAD.

MicroRNAs (miRNAs) are a class of small non‐coding RNAs of 20–22 nucleotides that regulate gene expression at the post‐transcriptional level and participate in a variety of biological pathways. Aberrantly expressed miRNAs have been found to be involved in various cancers, including LUAD 5, breast cancer 6, gastrointestinal cancer 7, ovarian cancer 8, and esophageal cancer 9, acting as oncogenes or antioncogenes 10, 11. In accumulated studies, aberrantly expressed miRNAs have been found to have potential diagnostic value for LUAD. Some well‐characterized miRNAs function as a diagnostic or prognostic biomarker for LUAD, such as *miRNA‐339‐5p, miRNA‐21, miRNA‐383, and miR‐7* 3, 12, 13.

The Cancer Genome Atlas (TCGA) is a central bank for multidimensional experimental cancer data that contributes to uncovering the molecular mechanisms of cancer. In the present study, we used TCGA to obtain and further analyze the miRNA and mRNA expression data of LUAD patients. The key miRNAs with great diagnostic value were identified and their roles in LUAD were further revealed by investigating the function of their target genes.

## Materials and methods

### Integrated profiles in TCGA

We downloaded the clinical data of LUAD patients from TCGA (http://tcga-data.nci.nih.gov/). rsem‐normalized mRNA expression profiles (Level 3‐IlluminaHiseq_RNASeqV2 data) and miRNA expression profile (Level 3‐IlluminaHiSeq‐miRNASeq data) of LUAD and adjacent normal tissues were downloaded from TCGA data portal (http://tcga-data.nci.nih.gov/) as well. Only patients who were diagnosed as LUAD histologically were included in the study.

### Identifying miRNAs and mRNAs to distinguish tumor from normal

Based on the read count of each sample, the differentially expressed miRNAs (DEmiRNAs) and mRNAs (DEmRNAs) in LUAD compared to normal tissues were calculated via the R‐bioconductor package deseq2 (http://bioconductor.org/packages/DESeq2/). We performed multiple comparisons by using the Benjamini and Hochberg method to obtain the false discovery rate (FDR). The threshold was FDR < 0.05 and |log_2_ fold change| > 4 for DEmiRNAs. In order to gain an overview of the characteristics of the miRNA expression profile, a heat‐map was further generated by hierarchical clustering analysis based on the normalized expression values of all DEmiRNAs using the r package (https://www.r-project.org/). For DEmRNAs, the threshold was defined as FDR < 0.05 and |log_2_ fold change| > 1.5.

### Statistics for classification and prediction

To identify optimal diagnostic miRNA biomarkers for LUAD, we performed a feature selection procedures as follows. Firstly, the importance value of each DEmiRNA was ranked according to the mean decrease in accuracy using the random forest analysis. Then, the optimal number of features was found by subsequently adding one DEmiRNA at a time in a top‐down forward‐wrapper approach. The diagnostic odds ratio (DOR) is defined as the ratio of the odds of the positivity of a diagnostic test among a diseased population relative to that in the non‐diseased population. DOR was assessed by using the support vector machine (SVM) at each increment, and the optimal diagnostic miRNA biomarkers for LUAD were identified.

These optimal DEmiRNAs with diagnostic value for LUAD were used to establish classification models including decision tree, the SVM model, and random forest to better distinguish LUAD and adjacent tissues. The decision tree model was established by using the rpart package (https://cran.r-project.org/web/packages/rpart/). The SVM model was established by using the e1071 package in r, and the random forest model was established by using the ‘randomforest’ package (https://cran.r-project.org/web/packages/randomForest/). The three kinds of classification models were compared by the average misjudgment rates of their 10‐fold cross‐validations. Diagnostic ability of classification prediction was evaluated by obtaining the area under a receiver operating characteristic (ROC) curve (AUC) and DOR. A heat‐map of the optimal DEmiRNAs with diagnostic value for LUAD was generated by hierarchical clustering analysis by using the r package.

### Identifying mRNA targets of miRNA

To uncover the potential roles of the identified optimal DEmiRNA diagnostic biomarkers, we predicted their targets. Considering the opposite trend in the expression of miRNA and their targets, we screened the significant negatively co‐expressed DEmiRNA–DEmRNA pairs by pairwise Pearson correlation coefficients. DEmiRNA–DEmRNA pairs with *P *<* *0.05 and *r* < 0 were defined as significant negative co‐expression pairs. The putative targets of DEmiRNAs were predicted by six bioinformatic algorithms (RNA22, miRanda, miRDB, miRWalk, PICTAR2 and Targetscan) of mirwalk2.0 (http://zmf.umm.uni-heidelberg.de/apps/zmf/mirwalk2/mir-mir-self.html). The targets recorded by more than four algorithms were served as target mRNAs of miRNAs. Moreover, the confirmed targets of miRNAs obtained by mirwalk2.0 were served as target mRNAs of miRNAs as well. Finally, significant negatively co‐expressed DEmiRNA–DEmRNA pairs overlapped with miRNA–target mRNA pairs were used to construct the DEmiRNA–DEmRNA interaction network by using cytoscape software (http://www.cytoscape.org/).

### Functional annotation of miRNA targets

To uncover the biological functions and detect the potential pathways of target DEmRNAs of DEmiRNAs, the online software genecodis was used to perform the functional annotation, including gene ontology (GO) classification (molecular functions, biological processes, and cellular component) and Kyoto Encyclopedia of Genes and Genomes (KEGG) pathway enrichment. Statistical significance was defined as FDR < 0.05.

### Confirmation of differentially expressed miRNAs and mRNAs

Ten LUAD tumor tissues and 10 normal adjacent tissues were obtained from 10 patients (P1–P10) who were diagnosed as LUAD based on pathological analysis. Patient characteristics are displayed in Table 1. We obtained written informed consent from the patients and approval from the ethics committee of Chinese PLA General Hospital. The study conformed to the Declarations of Helsinki. Total RNA was extracted with the TRIzol reagent (Invitrogen, Shanghai, China). One microgram RNA was used to synthesize cDNA using SuperScript® III Reverse Transcriptase (Invitrogen). Quantitative real‐time PCR (qRT‐PCR) was performed with Power SYBR® Green PCR Master Mix (Applied Biosystems, Carlsbad, CA, USA) in the ABI7500 real‐time PCR system (Applied Biosystems). And the reverse transcriptions of miRNAs were performed using the miScript II RT Kit (Qiagen, Hilden, Germany). qRT‐PCR was performed with miScript SYBR Green PCR Kit (Qiagen). Relative gene expression was analyzed using the $2^{-\Delta \Delta C_{t}}$ method. Statistical significance was assessed by one‐way ANOVA. The human 18S rRNA and U6 were used as endogenous controls for mRNA and miRNA expression in the analysis.

**Table 1:** Patient characteristics. P1–P10, patient 1–patient 10 with LUAD

Index	P1	P2	P3	P4	P5	P6	P7	P8	P9	P10
Age (years)	48	60	59	64	74	69	46	54	66	31
Male/female	Male	Female	Female	Female	Male	Male	Male	Male	Female	Male
BMI	24.3	18.3	25.1	26.6	21	22.3	23.7	31.1	24.4	19.7
TNM stage	T1BN0M0	T1AN0M0	T1BN0M0	T2AN0M0	T2AN0M0	T2AN2M0	T1AN0M0	T2AN0M0	T1AN0M0	T1N2M0
Family history	No	No	No	No	No	No	No	No	No	No
History of other cancers	No	No	No	No	No	No	No	No	No	No

## Results

### Identification of differentially expressed miRNAs and mRNAs between LUAD and adjacent tissues

The mRNA expression profiles of 533 patients with LUAD and 59 normal adjacent tissues were obtained. The median age of these 533 patients was 67 years. Female and male patients account for 53.7% and 46.3% of the population, respectively. The information of the TNM stage was as follows: T1N0M0–T1N2M1 (32.8%), T2N0M0–T2N2M1 (53.8%), T3N0M0–T3N2M1 (9.2%) and T4N0M0–T1N3M1 (3.6%). A total of 19 450 mRNAs were detected. mRNAs with read count value = 0 in more than 20% (*n* = 107) of tumors or in more than 20% (*n* = 12) of adjacent tissues were considered to be low/not expressed mRNAs.

The miRNA expression profiles of 519 patients with LUAD and 46 normal adjacent tissues were obtained. The median age of these 519 patients was 67 years. Female and male patients account for 53.6% and 46.4% of the population, respectively. The information of the TNM stage was as follows: T1N0M0–T1N2M1 (33.3%), T2N0M0–T2N2M1 (53.0%), T3N0M0–T3N2M1 (9.4%) and T4N0M0–T1N3M1 (3.7%). A total of 1881 miRNAs were detected. miRNAs with read count value = 0 in more than 60% (*n* = 311) of tumors or in more than 60% (*n* = 28) of adjacent tissues were considered to be low/not expressed miRNAs.

**Figure 1:**
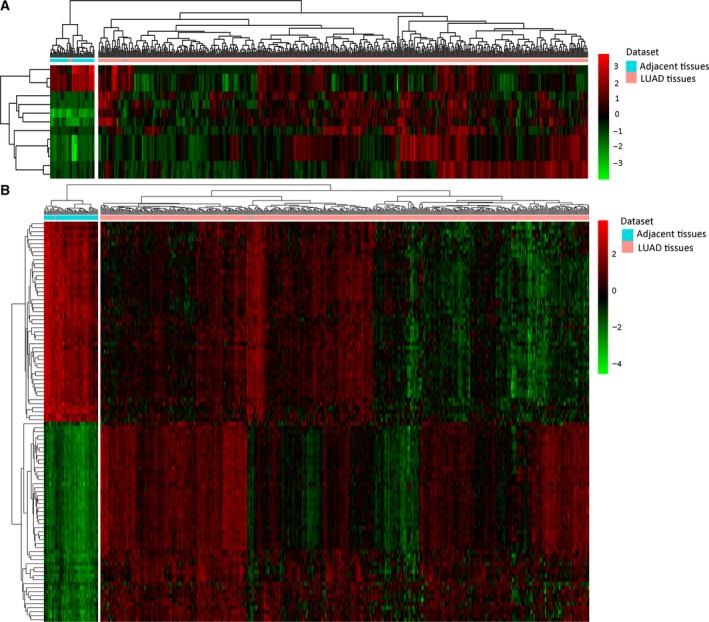
Hierarchical clustering analysis of DEmiRNAs and the top 100 DEmRNAs between LUAD and adjacent tissues. (A) DEmiRNAs; (B) the top 100 DEmRNAs. Rows and columns represent DEmiRNAs/DEmRNAs and tissue samples. The color scale represents the expression levels.

After filtering the low/not expressed mRNAs or miRNAs, 16 043 mRNAs and 559 miRNAs were retained for analysis, respectively. A total of 13 DEmiRNAs (three down‐regulated and 10 up‐regulated miRNAs) between LUAD and normal tissues were identified with FDR < 0.05 and |log_2_ fold change| > 4. A total of 2301 DEmRNAs (933 down‐regulated and 1368 up‐regulated mRNAs) between LUAD and normal tissues were identified with FDR < 0.05 and |log_2_ fold change| > 1.5. Hierarchical clustering analysis of DEmiRNAs and the top 100 DEmRNAs is displayed in Fig. 1A and B, respectively.

### Identification of optimal diagnostic miRNA biomarkers for LUAD

**Figure 2:**
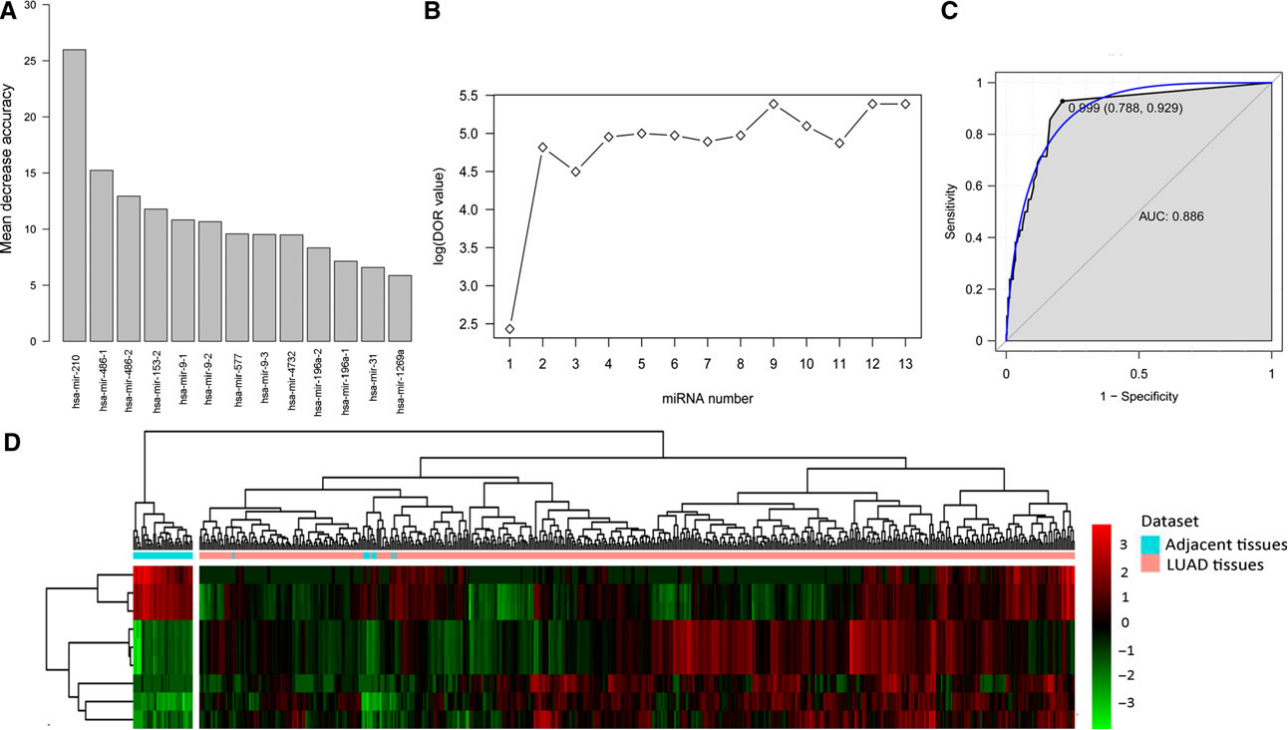
Identification of miRNA biomarkers for LUAD. (A) The variance rate of prediction accuracy for each DEmiRNA. (B) The variance rate of classification performance when increasing the number of the predictive miRNAs. (C) ROC results of combinations of nine miRNA biomarkers based on random forest classification model. (D) Hierarchical clustering analysis of the nine miRNA biomarkers between LUAD and adjacent tissues. Rows and columns represent DEmiRNAs and tissue samples. The color scale represents the expression levels.

To identify the optimal diagnostic miRNA biomarkers for LUAD, we performed random forest feature selection and classification (SVM, decision tree and random forest) procedures. All DEmiRNAs were ranked according to the standardized drop in prediction accuracy (Fig. 2A). Then, we compared the DOR increment for a specific number of miRNAs by subsequently adding one miRNA at a time in a top‐down forward‐wrapper approach. We found that the DOR of nine DEmiRNAs reached the highest point for the first time (Fig. 2B). Hence, these nine DEmiRNAs were defined as the optimal diagnostic miRNA biomarkers for LUAD (Table 2).

**Table 2:** The nine optimal diagnostic miRNA biomarkers for LUAD. Data were analyzed by Wald test

miRNA	Log_2_ fold change	*P*‐value	FDR	Regulation
*hsa‐mir‐210*	4.536466384	1.20E‐97	3.36E‐95	Up
*hsa‐mir‐486‐1*	−4.277102805	3.47E‐84	6.47E‐82	Down
*hsa‐mir‐486‐2*	−4.268035702	2.17E‐83	3.03E‐81	Down
*hsa‐mir‐153‐2*	4.204883454	1.11E‐52	3.26E‐51	Up
*hsa‐mir‐9‐1*	5.296508956	9.75E‐63	4.19E‐61	Up
*hsa‐mir‐9‐2*	5.303453252	7.22E‐63	3.67E‐61	Up
*hsa‐mir‐577*	5.026458962	1.40E‐39	2.11E‐38	Up
*hsa‐mir‐9‐3*	5.303960101	2.77E‐63	1.55E‐61	Up
*hsa‐mir‐4732*	−4.357779935	2.26E‐61	9.03E‐60	Down

These nine optimal DEmiRNAs with diagnostic value for LUAD were used to establish classification models including decision tree, SVM and random forest. The 10‐fold cross‐validation indicated that the misjudgment rate of SVM, decision tree and random forest was 5.12%, 1.59% and 1.42%, respectively. This result suggested that the random forest model, with the smallest average misjudgment rate, could effectively predict LUAD. Based on the classification model of random forest, the DOR of these nine DEmiRNAs was 7735.1; the ROC curves are displayed in Fig. 2C and hierarchical clustering analysis of the nine DEmiRNAs is displayed in Fig. 2D.

### miRNA–mRNA interactions in LUAD

**Figure 3:**
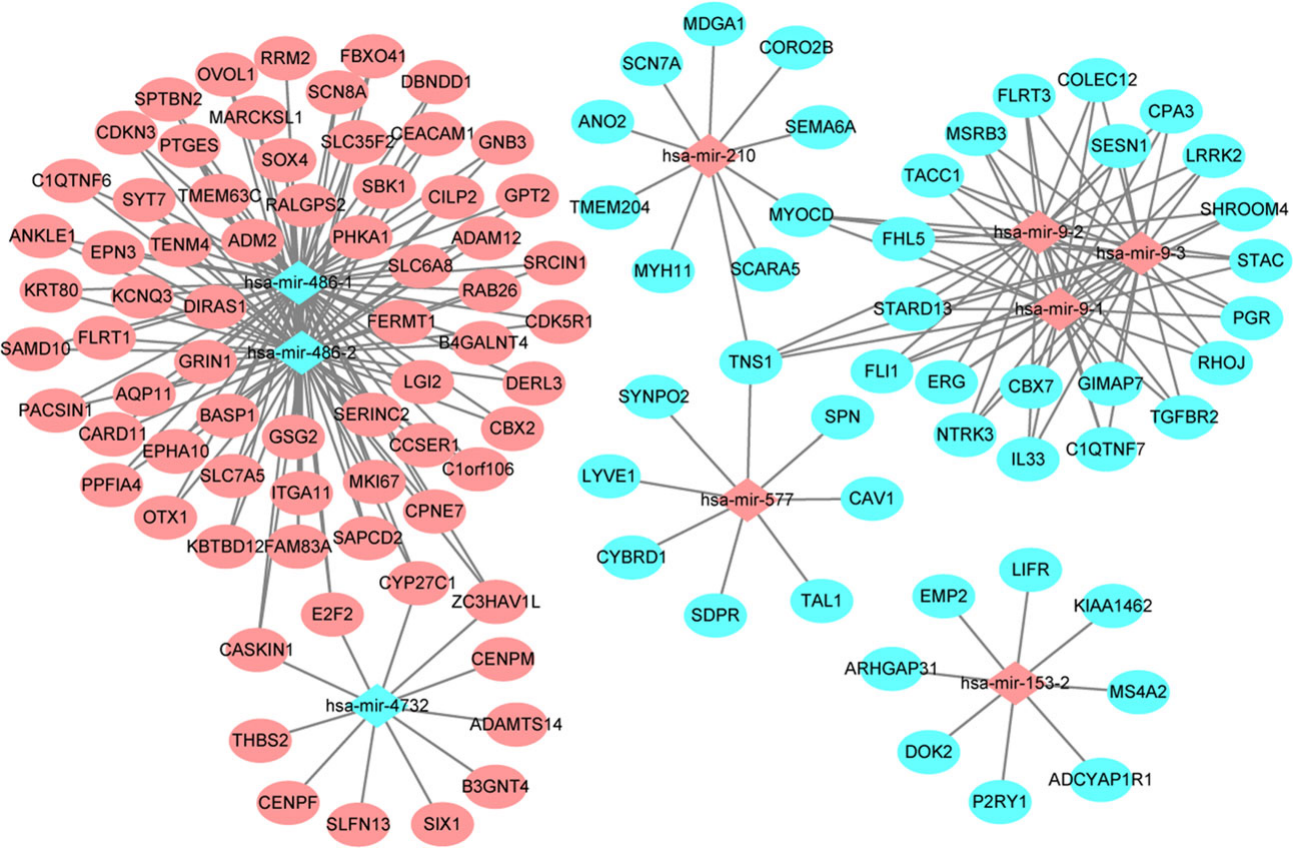
LUAD‐specific DEmiRNA–DEmRNA interaction network. The ellipses and rhombuses represent the DEmRNAs and DEmiRNAs, respectively. Red and blue represent up‐ and down‐regulation, respectively.

To better understand the role of these nine optimal DEmiRNAs in LUAD, their potential target was also analyzed. According to the miRNA–mRNA expression correlation analysis, we obtained 3341 DEmiRNA–DEmRNA pairs that were negatively correlated (*P* < 0.05, *r* < 0). Moreover, we obtained 409 miRNA–target mRNA pairs based on miRWalk including 362 miRNA–target mRNA pairs predicted by more than four algorithms and 47 confirmed miRNA–target mRNA pairs. After screening the overlapped miRNA–mRNA pairs among both these 409 miRNA–target mRNA pairs and 3341 negative DEmiRNA–DEmRNA co‐expression pairs, we obtained 232 DEmiRNA–target DEmRNA pairs including 116 DEmRNAs (70 up‐regulated and 46 down‐regulated DEmRNAs) and nine DEmiRNAs (six up‐regulated and three down‐regulated DEmiRNAs). Based on the DEmiRNA–target DEmRNA interaction network, *hsa‐mir‐486‐1* (degree = 63) and *hsa‐mir‐486‐2* (degree = 63) were two hub DEmiRNAs (Fig. 3).

### Functional enrichment analysis of miRNA targets

The 116 target DEmRNAs of the DEmiRNAs were used to conduct the GO and KEGG enrichment analysis. GO enrichment analysis (Table 3) revealed that the miRNA targets were significantly enriched in circulatory system development (*P* < 0.05), plasma membrane region (*P* < 0.05), caveola (*P* < 0.05) and regulation of synapse assembly (*P* < 0.05). KEGG pathway enrichment analysis (Table 3) showed that transcriptional misregulation in cancer (*P*‐value < 0.05) and central carbon metabolism in cancer (*P*‐value < 0.05) were two significantly enriched pathways.

**Table 3:** Functional annotation of targeted DEmRNAs of DEmiRNAs. Data were analyzed using the standard accumulative hypergeometric statistical test. BP, biological processes; CC, cellular components; MF, molecular functions

Category	ID	Description	Log (*P*‐value)	Symbols
GO (BP)	GO:0072359	Circulatory system development	−6.703660169	*CEACAM1*,* CAV1*,* EMP2*,* ERG*,* MYH11*,* NTRK3*,* SIX1*,* SOX4*,* TAL1*,* TGFBR2*,* THBS2*,* BASP1*,* FLRT3*,* TENM4*,* RHOJ*,* TMEM204*,* ADM2*,* STARD13*,* MYOCD*,* GRIN1*,* CDK5R1*,* SRCIN1*,* LRRK2*,* ADAM12*,* TNS1*,* FERMT1*,* IL33*
GO (CC)	GO:0098590	Plasma membrane region	−5.861204601	*ADCYAP1R1*,* CEACAM1*,* CAV1*,* EMP2*,* GRIN1*,* P2RY1*,* SPTBN2*,* TGFBR2*,* SLC7A5*,* SDPR*,* SYT7*,* FLRT3*,* PACSIN1*,* FERMT1*,* SHROOM4*,* CYBRD1*,* SRCIN1*,* LRRK2*
GO (CC)	GO:0005901	Caveola	−5.706471821	*ADCYAP1R1*,* CAV1*,* EMP2*,* TGFBR2*,* SDPR*,* LRRK2*,* CDK5R1*,* GSG2*,* SBK1*,* CEACAM1*,* DOK2*,* SRCIN1*,* FAM83A*,* DIRAS1*,* EPN3*,* CARD11*,* NTRK3*,* P2RY1*,* SPN*,* SERINC2*,* PHKA1*,* EPHA10*
GO (BP)	GO:0051963	Regulation of synapse assembly	−5.440592972	*GRIN1*,* NTRK3*,* SIX1*,* THBS2*,* FLRT3*,* FLRT1*,* CEACAM1*,* CAV1*,* TNS1*,* ITGA11*,* FERMT1*,* ARHGAP31*,* KIAA1462*,* TMEM204*,* SRCIN1*,* SYNPO2*,* SPTBN2*,* LRRK2*,* CDKN3*,* EMP2*,* TGFBR2*,* CDK5R1*,* MYOCD*,* DIRAS1*,* SERINC2*,* LIFR*,* DOK2*,* FAM83A*,* EPHA10*,* BASP1*,* TENM4*,* SOX4*,* TAL1*,* SPN*,* ADAMTS14*,* CILP2*,* P2RY1*,* PACSIN1*
GO (CC)	GO:0043005	Neuron projection	−5.242350102	*ADCYAP1R1*,* GNB3*,* GRIN1*,* KCNQ3*,* P2RY1*,* SCN8A*,* CDK5R1*,* SYT7*,* BASP1*,* FLRT3*,* FLRT1*,* TENM4*,* PACSIN1*,* SEMA6A*,* SRCIN1*,* LRRK2*,* AQP11*,* NTRK3*,* SPTBN2*,* DOK2*,* TAL1*,* RHOJ*,* SHROOM4*,* CEACAM1*,* CENPF*,* FERMT1*,* CYBRD1*,* OTX1*,* SIX1*,* THBS2*,* TACC1*,* ADM2*,* SPN*,* CAV1*
GO (BP)	GO:0007417	Central nervous system development	−4.874607656	*CENPF*,* GRIN1*,* NTRK3*,* OTX1*,* SIX1*,* SOX4*,* SPTBN2*,* TACC1*,* TAL1*,* TGFBR2*,* CDK5R1*,* BASP1*,* TENM4*,* SHROOM4*,* LRRK2*,* MDGA1*,* FLRT3*
GO (BP)	GO:0001570	Vasculogenesis	−4.476808317	*CEACAM1*,* CAV1*,* EMP2*,* TGFBR2*,* MYOCD*,* MYH11*,* P2RY1*,* SCN7A*,* SLC6A8*,* STAC*,* CDKN3*,* OVOL1*,* SOX4*,* SPN*,* PTGES*,* ADM2*,* SIX1*,* BASP1*,* TMEM204*,* LRRK2*
GO (CC)	GO:0044306	Neuron projection terminus	−4.379028359	*GRIN1*,* SYT7*,* FLRT3*,* FLRT1*,* PACSIN1*,* LRRK2*,* PPFIA4*,* RAB26*,* P2RY1*,* CDK5R1*,* SRCIN1*,* CEACAM1*,* CPA3*,* IL33*,* SLC6A8*,* EMP2*
GO (BP)	GO:0072001	Renal system development	−4.023285604	*CENPF*,* OVOL1*,* SIX1*,* SOX4*,* BASP1*,* MYOCD*,* LRRK2*,* AQP11*,* ERG*,* MYH11*,* NTRK3*,* TGFBR2*,* FLRT3*,* TENM4*,* CAV1*,* ADAM12*,* ITGA11*,* TMEM204*,* CDK5R1*,* STARD13*,* CEACAM1*
GO (CC)	GO:0009986	Cell surface	−4.021192798	*ADCYAP1R1*,* CEACAM1*,* EMP2*,* MS4A2*,* GRIN1*,* KCNQ3*,* P2RY1*,* SPN*,* TGFBR2*,* TNS1*,* PPFIA4*,* AQP11*,* SCARA5*,* CAV1*,* MKI67*,* NTRK3*,* PGR*,* SIX1*,* PTGES*,* COLEC12*,* LRRK2*,* SCN7A*,* CYBRD1*,* SOX4*,* RRM2*,* C1QTNF6*
GO (MF)	GO:0003779	Actin binding	−3.668606032	*CEACAM1*,* MYH11*,* SPTBN2*,* TNS1*,* CORO2B*,* SHROOM4*,* MARCKSL1*,* LRRK2*,* SYNPO2*,* CAV1*,* EMP2*,* NTRK3*,* CDK5R1*,* PACSIN1*,* RHOJ*,* CENPF*,* MS4A2*,* DOK2*,* ITGA11*,* ADM2*,* GNB3*,* COLEC12*,* FERMT1*,* SRCIN1*,* ADAMTS14*
GO (CC)	GO:0043235	Receptor complex	−3.62673173	*ADCYAP1R1*,* CEACAM1*,* GRIN1*,* LIFR*,* NTRK3*,* TGFBR2*,* ITGA11*,* CARD11*,* SPN*,* IL33*,* EMP2*,* CAV1*,* SIX1*,* SOX4*,* MARCKSL1*,* MYOCD*,* FLI1*,* TAL1*,* ANKLE1*,* SLC7A5*,* DOK2*,* CDK5R1*,* SRCIN1*
GO (BP)	GO:0042063	Gliogenesis	−3.613567247	*NTRK3*,* P2RY1*,* SOX4*,* TAL1*,* CDK5R1*,* TENM4*,* IL33*,* SIX1*
GO (MF)	GO:0001077	Transcriptional activator activity, RNA polymerase II core promoter proximal region sequence‐specific	−3.476014856	*ERG*,* FLI1*,* OTX1*,* PGR*,* SIX1*,* SOX4*,* MYOCD*,* OVOL1*,* E2F2*,* TAL1*,* BASP1*,* CEACAM1*,* CAV1*,* TGFBR2*,* SEMA6A*,* LRRK2*,* CENPF*,* CBX2*,* ANKLE1*
GO (MF)	GO:0005516	Calmodulin binding	−3.378339299	*GRIN1*,* KCNQ3*,* MYH11*,* PHKA1*,* SYT7*,* MARCKSL1*
GO (BP)	GO:0051051	Negative regulation of transport	−3.338324909	*CEACAM1*,* CAV1*,* P2RY1*,* SOX4*,* PACSIN1*,* SRCIN1*,* IL33*,* DERL3*,* LRRK2*,* SYT7*,* RAB26*,* CARD11*
GO (BP)	GO:0003179	Heart valve morphogenesis	−3.127090085	*ERG*,* SOX4*,* TGFBR2*,* TAL1*,* MDGA1*,* SIX1*,* MYOCD*,* E2F2*,* ANKLE1*,* OTX1*,* FLRT3*,* TENM4*,* CEACAM1*,* STARD13*,* MKI67*
GO (BP)	GO:1903034	Regulation of response to wounding	−3.000153384	*CEACAM1*,* CAV1*,* NTRK3*,* TGFBR2*,* SCARA5*,* ERG*,* SYT7*,* P2RY1*,* AQP11*,* LYVE1*,* FLRT3*,* PACSIN1*,* COLEC12*,* GNB3*,* SIX1*,* CDK5R1*,* SESN1*,* LRRK2*,* SOX4*,* GRIN1*,* SPN*,* IL33*,* RAB26*,* FLRT1*,* FLI1*,* SPTBN2*,* DIRAS1*,* EMP2*
GO (BP)	GO:0048013	Ephrin receptor signaling pathway	−2.989818807	*GRIN1*,* NTRK3*,* CDK5R1*,* EPHA10*,* TGFBR2*,* ADCYAP1R1*,* LIFR*,* MDGA1*
GO (BP)	GO:0051899	Membrane depolarization	−2.798135634	*CAV1*,* SCN7A*,* SCN8A*,* LRRK2*,* ADCYAP1R1*,* GRIN1*,* KCNQ3*,* P2RY1*,* STAC*,* SYT7*,* ANO2*,* TMEM63C*,* CEACAM1*,* SLC6A8*,* SLC7A5*,* SERINC2*,* SCARA5*
KEGG pathway	ko04713	Circadian entrainment	−1.885359167	*ADCYAP1R1*,* GNB3*,* GRIN1*
KEGG pathway	hsa05202	Transcriptional misregulation in cancer	−1.867247226	*ERG*,* FLI1*,* SIX1*,* TGFBR2*
KEGG pathway	ko05030	Cocaine addiction	−1.587657877	*GRIN1*,* CDK5R1*
KEGG pathway	hsa05212	Pancreatic cancer	−1.374868302	*E2F2*,* TGFBR2*
KEGG pathway	hsa05230	Central carbon metabolism in cancer	−1.362710373	*NTRK3*,* SLC7A5*
KEGG pathway	hsa04115	p53 signaling pathway	−1.316099311	*RRM2*,* SESN1*

### ROC analyses

By using the proc package in the r language, we performed ROC analyses to assess the diagnostic value of DEmiRNAs and their targets. The AUC under binomial exact confidence interval was calculated and a ROC curve was generated.

### ROC curve analysis

**Figure 4:**
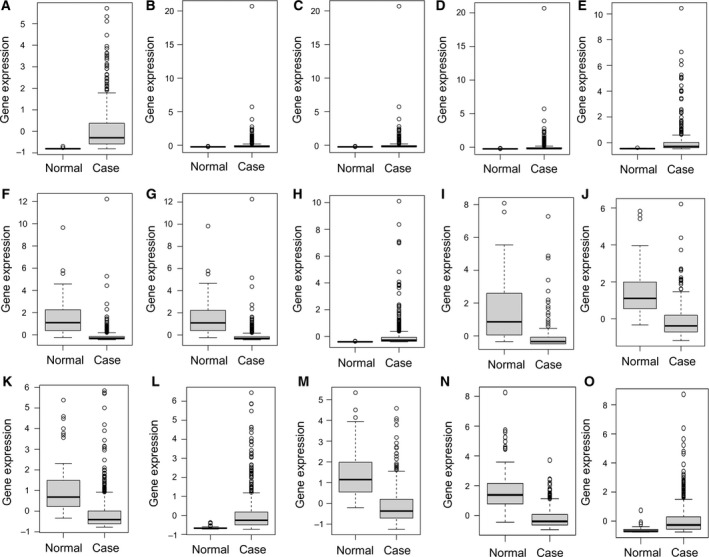
The expression levels of DEmiRNAs and DEmRNAs between LUAD and adjacent tissues. The *x*‐axes represent normal (LUAD) and control (adjacent tissues) groups. The *y*‐axes represent gene expression levels. (A) *hsa‐mir‐210*, (B) *hsa‐miR‐9‐1*, (C) *hsa‐miR‐9‐2*, (D) *hsa‐miR‐9‐3*, (E) *hsa‐mir‐153‐2*, (F) *hsa‐mir‐486‐1*, (G) *hsa‐mir‐486‐2*, (H) *hsa‐mir‐577*, (I) *hsa‐mir‐4732*, (J) *FLI1*, (K) *NTRK3*, (L) *SLC7A5*, (M) *TGFBR2*, (N) *ERG* and (O) *SIX1*.

**Figure 5:**
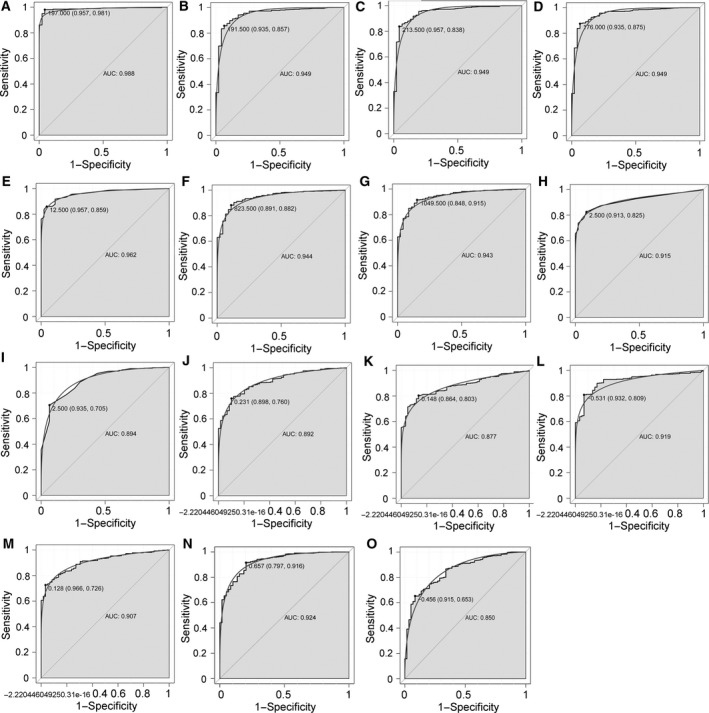
The ROC curves of selected DEmiRNAs and DEmRNAs between LUAD and adjacent tissues. The *x*‐axes show 1 − specificity (the proportion of false positives) and *y*‐axes show sensitivity (the proportion of true positives). (A) *hsa‐mir‐210*, (B) *hsa‐miR‐9‐1*, (C) *hsa‐miR‐9‐2*, (D) *hsa‐miR‐9‐3*, (E) *hsa‐mir‐153‐2*, (F) *hsa‐mir‐486‐1*, (G) *hsa‐mir‐486‐2*, (H) *hsa‐mir‐577*, (I) *hsa‐mir‐4732*, (J) *FLI1*, (K) *NTRK3*, (L) *SLC7A5*, (M) *TGFBR2*, (N) *ERG* and (O) *SIX1*.

We performed ROC curve analyses and calculated the AUC to assess the discriminatory ability of these nine DEmiRNAs and six differentially expressed targets between LUAD and adjacent tissues. The expression levels of these nine DEmiRNAs and six differentially expressed targets between LUAD and adjacent tissues are displayed in Fig. 4. The AUC of nine DEmiRNAs and six targets including *hsa‐mir‐210* (0.988), *hsa‐mir‐9‐1* (0.949), *hsa‐mir‐9‐2* (0.949), *hsa‐mir‐9‐3* (0.949), *hsa‐mir‐153‐2* (0.962), *hsa‐mir‐486‐1* (0.944), *hsa‐mir‐486‐2* (0.943), *hsa‐mir‐577* (0.915), *hsa‐mir‐4732* (0.894), *FLI1* (0.892), *NTRK3* (0.877), *SLC7A5* (0.919), *TGFBR2* (0.907), *ERG* (0.924) and *SIX1* (0.850) was more than 0.85 (Fig. 5).

### Confirmation of differentially expressed miRNAs and mRNAs

**Figure 6:**
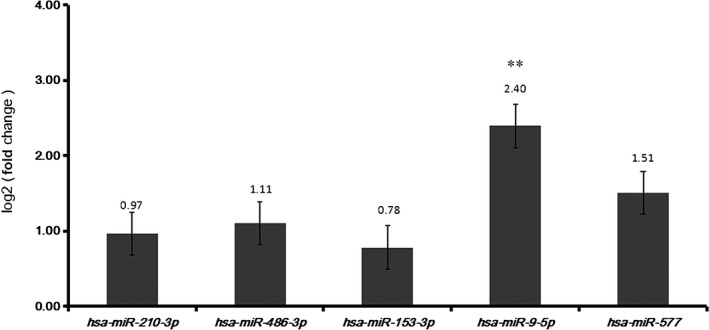
qRT‐PCR results for DEmiRNAs between LUAD and adjacent tissues. Statistical significance was assessed by one‐way ANOVA. The error bars indicate SD. *n* = 3. The *x*‐axis and *y*‐axis represent DEmiRNAs and log fold change, respectively. **P < 0.01.

Quantitative real‐time‐PCR of 10 pairs of LUAD and the adjacent tissues were used to verify the expression of five DEmiRNAs (*hsa‐mir‐210‐3p*,* hsa‐mir‐486‐3p*,* hsa‐mir‐153‐3p*,* hsa‐mir‐9‐5p*,* hsa‐mir‐577*). Based on TCGA, *hsa‐mir‐486‐3p* was down‐regulated while the other four DEmiRNAs were up‐regulated in LUAD compared to adjacent tissues. According to the qRT‐PCR results, all the five DEmiRNAs were up‐regulated, which was consistent with the results for TCGA, generally (Fig. 6).

## Discussion

Lung adenocarcinoma is the most frequent subtype of lung cancer, with high global incidence and mortality 14. It is essential to explore accurate and specific biomarkers for it. Accumulated evidence indicates that miRNAs play crucial roles in the progress of LUAD. However, research into the diagnostic and prognostic value of miRNAs is currently in its infancy.

In this study, we performed genome‐wide analysis of miRNAs and mRNAs in a large number of patients with LUAD from TCGA. Altered miRNA expression patterns were found between LUAD and adjacent tissues, suggesting a potential diagnostic role for miRNA for LUAD. To identify the optimal LUAD‐specific miRNA biomarkers, we searched for combinations of miRNAs among the 13 DEmiRNAs whose expression pattern may distinguish LUAD from adjacent tissues with the highest DOR by using random forest feature selection. A nine‐miRNA combination including three down‐regulated miRNAs (*hsa‐mir‐486‐1*,* hsa‐mir‐486‐2*, and *hsa‐mir‐4732*) and six up‐regulated miRNAs (*hsa‐mir‐210*,* hsa‐mir‐153‐2*,* hsa‐mir‐9‐1*,* hsa‐mir‐9‐2*,* hsa‐mir‐577*, and *hsa‐mir‐9‐3*) were served as optimal LUAD‐specific miRNA biomarkers. The 10‐fold cross‐validation of three kinds of models (decision tree, SVM, and random forest) suggested that the random forest model, with the smallest average misjudgment rate of 1.42%, could effectively predict LUAD. Moreover, the AUC of all these nine DEmiRNAs in LUAD was more than 0.85. Taken together, our study has demonstrated the feasibility and potential diagnostic value of these nine miRNA biomarkers in LUAD.

Among them, *hsa‐mir‐486‐1* and *hsa‐mir‐486‐2* were two hub DEmiRNAs based on the DEmiRNA–DEmRNA interaction network, suggesting their importance in LUAD. Down‐regulation of mir‐486 has been demonstrated to be involved in various cancers including LUAD 15, 16, 17. Moreover, mir‐486 was reported to be cytotoxic in A549 LUAD cells by repressing the expression of bone morphogenetic protein‐2 reporter gene 18. *Hsa‐mir‐153* was found to be down‐regulated in NSCLC tissues and cell lines; it inhibits migration and invasion of NSCLC by targeting *ADAM19* 19 and protein kinase B (*AKT*) 20. These three miRNAs were speculated to serve as tumor suppressors in LUAD.

The other six DEmiRNAs were all up‐regulated in LUAD compared to adjacent tissue based on our study, which suggested their potential oncogenic roles in LUAD. *Hsa‐mir‐210* was the most up‐regulated miRNA in LUAD compared to adjacent tissue based on our study, which was consistent with previous studies 21, 22. Moreover, up‐regulation of *hsa‐mir‐210* was found to be closely associated with distant metastases of LUAD 21. Both *hsa‐mir‐9* and *hsa‐mir‐577* were also found to be up‐regulated in human NSCLC tissues and cell lines compared to normal lung tissues 16, 23, 24. *Hsa‐mir‐9* was reported to play an oncogenic role in the proliferation, invasion, and migration of NSCLC cells through mechanisms such as regulating SOX7 23, eukaryotic translation initiation factor 5A2 25, FoxO1 26, epithelial‐to‐mesenchymal transition and the signal transduction pathway 27. *Hsa‐mir‐577* could play a role in the cell proliferation of esophageal squamous cell carcinomas and glioblastoma multiforme by regulating a tumor‐associated antigen, testis specific 10 28, and the Wnt signaling pathway 29, respectively. However, the precise role of mir‐577 in LUAD remains unknown.

Although there is no study that reports an association between *hsa‐mir‐4732* and LUAD, *hsa‐mir‐4732* was indicated to be associated with breast cancer 30. Our study is the first to report the down‐regulation of *hsa‐mir‐4732* in LUAD compared to adjacent tissues.

To deeply research the biological functions of these DEmiRNAs in LUAD, we conducted a functional annotation of the target DEmRNAs of these DEmiRNAs. Two significantly enriched pathways in LUAD and their related DEmRNAs, namely transcriptional misregulation in cancer (*TGFBR2*,* FLI1*,* ERG* and *SIX1*) and central carbon metabolism (*NTRK3* and *SLC7A5*), were speculated to play key roles in LUAD regulated by DEmiRNAs. *TGFBR2* was a putative tumor suppressor gene in the TGF‐β signaling pathway. Loss of *Tgfbr2* in a K‐ras‐induced LUAD mouse model has been found to induce a highly invasive phenotype associated with lymph node metastasis and reduced survival. In our study, *TGFBR2* was down‐regulated in LUAD tissues compared to adjacent tissues, which was in agreement with previous study. Both *FLI1* and *ERG* are members of erythroblast transformation‐specific oncogene family. *FLI1* was down‐regulated in squamous lung cancer 31 and could up‐regulate *TGFBR2* in LUAD and might be a potential regulator of LUAD 32. Overexpressed *ERG* variant 2 was found in lung tumors compared to adjacent tissues, which suggested that *ERG* might exert an oncogenic effect in lung tumors through functions encoded by variant 2 33. Aberrantly methylated *NTRK3* has been demonstrated to be involved in various cancers including colorectal cancer and lung cancer 34, 35. Poor survival in lung cancer was closely associated with *NTRK3* 36. *TGFBR2*,* FLI1*,* ERG* and *NTRK3* were shared DEmRNAs of *hsa‐mir‐9‐1*,* hsa‐mir‐9‐2* and *hsa‐mir‐9‐3*, which suggested that mir‐9 might play a crucial role in LUAD by regulating these four lung cancer‐related genes.

The *SIX* family was demonstrated to play crucial roles in the tumorigenesis of NSCLC and was associated with the prognosis of patients with NSCLC 37. As a member of the *SIX* family, *Six1* was found to be up‐regulated in LUAD and contributes to preinvasive‐to‐invasive adenocarcinoma progression by inducing epithelial–mesenchymal transition and nuclear atypia 38. The protein SLC7A5 is part of a two‐protein complex with SLC3A2 and the gene was hypermethylated and its expression increased in lung cancer 35. Moreover, *SLC7A5* was also reported to be differentially expressed between adenocarcinoma and squamous cell lung cancers 39, 40. Both *SLC7A5* and *Six1* were target genes of *hsa‐mir‐486* and *hsa‐mir‐4732*, respectively. We speculate that *hsa‐mir‐486*–*SLC7A5* and *hsa‐mir‐4732*–*Six1* interactions might also be involved in the pathogenesis of LUAD. Moreover, the ROC analysis of the present study indicated that all these six targets have great diagnostic value for LUAD and might be potential biomarkers of LUAD as well.

In conclusion, our study identified nine DEmiRNAs that serve as potential biomarkers of LUAD. Functional annotation of their target DEmRNAs in LUAD provided new clues for exploring their precise roles in LUAD. Further validation studies in prospective datasets are needed to test the predictive power for diagnosis before this is applied clinically.

## Conflict of interest

The authors declare no conflict of interest.

## Author contributions

Z‐PR and X‐BH drafted the manuscript; X‐DT and J‐TG participated in data collection; L‐BZ, Z‐QX and J‐QD carried out the data analysis; S‐WZ, J‐YP, and X‐YC had significant roles in the study design and manuscript review. All authors read and approved the final manuscript.
